# Cognitive disorder and associated factors among pregnant women attending antenatal service at Dilla University Referral Hospital, 2022

**DOI:** 10.3389/fgwh.2023.1061626

**Published:** 2023-05-19

**Authors:** Chalachew Kassaw, Tiruwork Wale, Misrak Negash, Kiber Temesgen, Birhanie Mekuriaw, Omega Tolessa, Elias Nigusu Abdisa, Yigrem Ali Chekol, Getinet Ayano, Tamrat Anbesaw

**Affiliations:** ^1^ Department of Psychiatry, Dilla University, Dilla, Ethiopia; ^2^ Department of Nursing, Wolkite University, Wolkite, Ethiopia; ^3^ Department of Comprehensive Nursing, Wachemo University, Hossana, Ethiopia; ^4^ Department of Psychiatry, Injibara University, Injibara, Ethiopia; ^5^ Research and Training Department, Amanuel Mental Specialized Hospital, Addis Ababa, Ethiopia; ^6^ School of Public Health, Curtin University, Perth, WA, Australia; ^7^ Department of Psychiatry, College of Medicine and Health Science, Wollo University, Dessie, Ethiopia

**Keywords:** Antenatal Care, Cognitive, pregnancy, Dilla, Ethiopia

## Abstract

**Introduction:**

Cognition is defined as the mental activity or process of learning information and understanding through reason, experience, and the senses. In Sub-Saharan African nations like Ethiopia, such assessments of a pregnant mother's mental health during antenatal care are uncommon procedures. Instead, there is a greater focus on the physical well-being of the woman and her fetus. As a result, this study aimed to evaluate the cognitive deficits and related factors in a pregnant women attending an antenatal care service.

**Methods:**

This hospital-based cross-sectional study included 415 pregnant women who were receiving antenatal care at Dilla University Referral Hospital in Dilla, Gedeo Zone, Ethiopia. In this study, respondents were chosen using systematic random sampling, and study participants were interviewed using administered questions to gather pertinent data. This study used the OSLO Social Support Scale, the Alcohol, Smoking, and Substance Involvement Screening Test, and the Mini-Mental Status Examination to assess the social support, cognitive status, and current substance use history of a respondent. Descriptive statistics including frequencies, graphs, and percentages were used to describe the results. A logistic regression analysis was conducted to determine the connection between independent factors and the outcome variable at a 95 percent confidence level and *p* < 0.05.

**Result:**

Among all respondents who came for antenatal care visits, only 24 (5.8%) were unmarried (single, divorced, widowed). The mean age of respondents was 26 years old and 155 (37.3%) had attended secondary school. Variables such as strong social support [0.11 (0.03–0.23), *p* < 0.02], being a follower of orthodox religion [0.24 (0.12–0.39), *p* < 0.04], ≥5,000 Ethiopian birr monthly income [0.28 (0.17–0.48), *p* < 0.02], age >26 years old [1.23 (1.14–2.54), *p* < 0.04], unplanned pregnancy [2.78 (1.45–4.32), *p* < 0.02], and rural residence [3.90 (2.23–7.34), *p* < 0.04] were significantly associated with cognitive impairment at 95% confidence interval and a *p*-value <0.05.

**Conclusion:**

This study found that pregnant women who attended antenatal care experienced a significant reduction in cognitive disorders. Additionally, this study revealed adjustable factors such as unwanted pregnancy, social support, and religiosity. It is preferable to check a pregnant woman's cognitive condition at antenatal services and to follow-up on each additional visit.

## Introduction

The term “cognition” identifies a set of mental functions that are included in the receiving, processing, interpreting, and retrieving of information (1). Cognitive abilities are essential to a person's entire development because they involve many of the brain's fundamental processes, including thinking, reading, learning, retaining knowledge, and paying attention. These processes are used to solve problems, recall tasks, and make judgments (2). Globally, the prevalence of cognitive impairment ranges from 5.1% to 41%, with a median of 19.0%, and the incidence of cognitive impairment ranges from 22% to 76.8%, with a median of 53.97 per 1,000 person-year (3).

The term “pregnancy” is used to describe the time when a fetus develops inside a woman's uterus or womb from the last menstrual cycle to delivery. The average pregnancy lasts roughly 40 weeks, or just over 9 months (4). Worldwide, approximately one-fourth of adolescent women have become pregnant and in Africa, the percentage of adolescent pregnancy is 18.8%, with 19.3% of reported cases in Sub-Saharan Africa and 21.5% in Eastern Africa (5).

Many women go through a period of cognitive shift known as the “pregnancy brain” during pregnancy and the postpartum period. Mental fogginess and memory issues are the symptoms that women at these reproductive stages report experiencing the most (6). Additionally, it involves a general lack of emotional stability and self-control as well as an aggravating loss of declarative memory, which includes forgetting names and important faces (7).

The prevalence of cognitive impairment among pregnant women has been reported in several countries including northern India (23.73 ± 4.69) (8), Indonesia (81%) (9), and Nigeria (58.3%) (10). The contributing factors for cognitive problems during pregnancy are a maternal occupation, a change in thyroid functions (hypothyroxinemia) (11), second and third-trimester gestational age (12), incomplete pregnancy (13), co-morbid medical conditions (14), physical exercise (15), and personality characteristics differences (16). The health of the mother and the unborn child may suffer as a result of cognitive impairment during pregnancy, which could lead to an undesirable pregnancy and unhealthy offspring. Experiencing a cognitive disorder while pregnant causes changes to hormones such as cortisol, which have a direct impact on the baby's normal brain development (17). According to imaging studies, a pregnant woman's mental changes are also responsible for a baby having an active amygdala in the womb, which is connected to emotional problems like anxiety and sadness (18). Furthermore, mental instability during pregnancy has an impact on a baby's hippocampal growth and brain chemistry, both of which are crucial for learning and emotional expression (19). Managing cognitive changes during pregnancy requires regular eating habits and physical activity, maintaining stable relationships with friends and family, and abstaining from the use of narcotics and psychoactive substances (20).

Despite the importance of the health of a mother and her fetus, there are insufficient studies done in East Africa, including Ethiopia. As a consequence, the purpose of this study is to determine the prevalence of cognitive impairment and its factors among pregnant women who would get ANC follow-up service at Dilla University Referral Hospital. This study aims to identify knowledge gaps, demonstrate the severity of the issue in research areas, assist in creating plans and interventions that are appropriate for pregnant women and the cognitively impaired, and provide knowledge to health professionals so they can develop efficient strategies.

## Methods and materials

### Study area

The study was conducted at Dilla University Referral Hospital (DURH). DURH has five wards, namely medical (39 beds), surgical (26 beds), obstetrics/gynecology (9 beds), pediatrics (18 beds), and psychiatry (12 beds). The hospital has one psychiatry outpatient department, three medical outpatient departments, one surgical outpatient department, one pediatric outpatient department, and one antenatal care (ANC) follow-up service. ANC is a maternal healthcare service provided by skilled healthcare professionals aimed at risk identification, prevention, management of pregnancy-related or concurrent disease, and health education and health promotion.

### Study design and period

An institutional-based cross-sectional study was conducted from May 12 to June 30, 2022.

### Study populations

This study enrolled all pregnant women who were attending antenatal follow-up at DURH in Dilla Town Public Health Institution. All pregnant mothers who came to DURH for an ANC follow-up visit during the time of the data collection period were the study population.

### Eligibility criteria

All pregnant women aged 18 years and above were included in this study, whereas participants who were critically ill or had difficulty communicating were excluded from the study.

### Sample size determination

The sample size was determined using a single population proportion formula $\left(n\;=\;(Z_{\alpha }/2{)}^{2}p(1-p)/d^{2}\right)$ by an assumption of *p*^ ^=^ ^50%, based on assumptions of unknown proportion with 95% confidence interval and 5% margin of error. By assuming a 10% non-response rate, the total sample size was computed to be (1.96)^2  ^× 0.5(1–0.5)/(0.5)^2 ^= 384 + 38(10%) = 422.

### Sampling procedure

All eligible pregnant women attending at Dilla University were eligible to participate. A systematic random sampling technique was used to select the study participants. On average, a total of 1,100 participants came for ANC visits in each month. A regular interval (K) was calculated as 1100^ ^÷^ ^422^ ^=^ ^2.60–3. Therefore, every 3rd patient from the list of appointments was selected.

### Data collection procedures and instruments

After the data was gathered utilizing the interview-administered data collection method, the data was recorded using the epi-collect software. Socio-demographic data, obstetric and maternal factors, substance-related factors, clinical issues, social factors, and cognitive impairment were all questioned by the data collectors. The information was gathered from participants using a questionnaire that was translated into Amharic and Gedeoffa language.

**Standard Mini-Mental State Exam (SMMSE–30**) is a tool used to assess cognitive impairment and includes components for orientation, attention, registration, computation, recall, and language. The administration time is between 10 and 15 min. The Mini-Mental State Examination (MMSE) is graded on a scale of 0–30, with 0–9 indicating severe cognitive impairment, 10–19 indicating moderate cognitive impairment, 20–24 indicating mild cognitive impairment, and >24 indicating no cognitive impairment. The patient's native language, educational attainment, and culture are considered when calculating the results of the mental status evaluation (25.26.27). A score of 22/30 was deemed to indicate cognitive impairment for participants with the educational levels of being unable to read or write and having not completed grade 8. Participants with an educational level of grade 9 were regarded to have cognitive impairment if their scores were less than 24/30. The MMSE had a sensitivity of 81% (95% CI, 78%–84%) and specificity of 89% (95% CI, 87%–92%) (17).

**The Alcohol, Smoking, and Substance Involvement Screening Test (ASSIST-3.0)** was used to evaluate individuals' current use of alcohol, tobacco, chewing khat, and cannabis. It was created by the World Health Organization (WHO) to identify the use of psychoactive substances and associated issues in patients receiving primary care. Participants were classified as current substance users if they had used any psychoactive substance in the last three months (18).

**The Oslo Social Support Scale (OSS-3)** is a quick test of social functioning, regarded as one of the finest indicators of mental health. It includes several social support fields such a counting a number of helping groups. It measures other closeness, interest, and concern, and assistance in the real day-to-day activities. The OSS-3 results ranged from 3 to 14 on a scale; 3–8 is considered poor, 9–11 indicates moderate support; 12–14 indicates strong support (19).

### Measurement

The outcome variable (dependent variable) was “cognitive impairment” among pregnant women. Thus, the independent variables used for multiple logistic regression analysis were age, religion, marital status, educational status, number of family members, residence, income, and occupation. Clinical factors in this study were previous history of mental illness, physical and psychological factors, abortion, stillbirth, live birth, neonatal death, pregnancy planned, total number of pregnancies, gestational age, number of antenatal care visits, current use of khat chewing, alcohol intake, and cigarette smoking.

### Data quality assurance

The pretest was done on participants with 5% of the sample size in Yirgga Cheffe hospital. Data collectors and supervisors were trained on the data collection tool and sampling techniques. Supervision was held regularly during the data collection period by both the researcher and supervisors. Written informed consent was taken from all respondents before the actual data collection period. The wholeness and consistency of the data were cross-checked and cleaned manually.

### Statistical analysis and processing

Epi-collect software was used to enter the data, which was then transferred to SPSS version 20 for additional analysis and cleaning. The results were presented using frequency, proportion, and descriptive techniques. To identify independently associated factors for cognitive impairment, binary logistic regression was used to determine the association between different factors and the outcome variable, and then all independent variables with a *p*-value of <0.25 were entered in the final model (multivariable logistic regression). The model fitness was examined using the Hosmer and Lemeshow goodness-of-fit test. At the appropriate 95% confidence interval, factors with a *p*-value of 0.05 or lower were statistically significantly associated with cognitive impairment.

## Results

### Socio-demographic data

This study included a respondents with 98% response rate. Among all respondents who came for antenatal visits, 24 (5.8%) were unmarried (single, divorced, widowed). The median income of the respondents was 5,000 Ethiopian birr and 155 (37.3%) had attended secondary school. Out of all respondents, 396 (95.4%) were from a rural area and 87 (20.9%) had government worker husbands (Table 1).

**Table 1:** Socio-demographic characteristics of respondents attending antenatal services at Dilla University Referral Hospital, Dilla, 2022, (*N* = 415).

Variable	Frequency	Percentage
**Age**		
<26	269	64.8%
≥26	146	35.2%
**Marital status**		
Married	391	94.2%
Unmarried	24	5.8%
**Religion**		
Protestant	201	48.4%
Orthodox	159	38.3%
Muslim	55	13.3%
**Monthly income**		
<5,000 ETB	238	57.3%
≥5,000 ETB	177	42.7%
**Educational status**		
Unable to read and write	60	14.5%
Primary	138	33.3%
Secondary	155	37.3%
College and above	62	14.9%
**Residence**		
Urban	396	95.4%
Rural	19	4.6%
**Husband occupation**		
Private	328	79.1%
Government	87	20.9%
**Family size**		
<3 size	246	59.3%
≥3 size	169	40.7%

### Pregnancy-related variables

Out of the respondents who came for antenatal care, 344 (82.9%) of them had planned pregnancy, and 29 (7%) had current pregnancy complications. The majority of the participants 183 (44.1%) were the second trimester and 47 (11.3%) of them had a history of abortion (Table 2).

**Table 2:** Pregnancy-related characteristics of respondents attending antenatal service at Dilla University Referral Hospital, Dilla, 2022, (*N* = 415).

Variable	Frequency	Percentage
**Planned pregnancy**		
Yes	344	82.9%
No	71	17.1%
**Abortion**		
Yes	47	11.3%
No	368	88.7%
**Current pregnancy complication**		
Yes	29	7.0%
No	386	93.0%
**Gestational age**		
First	51	12.3%
Second	183	44.1%
Third	181	43.6%
**Past history of pregnancy complications**		
Yes	59	14.2%
No	356	85.8%
**Neonatal death**		
Yes	16	3.9%
No	399	96.1%
**Number of pregnancies**		
<2	121	29.2%
≥2	294	70.8%

### Psychosocial variables

Out of the total respondents, 4 (1%) had a history of rape and 52 (12.5%) had a history of the loss of a loved one. Only 24 (5.8%) of respondents had reported a history of physical, psychological, or sexual violence and 2 (0.5%) had been diagnosed with mental illness (Table 3).

**Table 3:** Psychosocial characteristics of respondents attending antenatal service at Dilla University Referral Hospital, Dilla, 2022, (*N* = 415).

Variable	Frequency	Percentage
**History rape**		
Yes	4	1.0%
No	411	99.0%
**Loss of a loved one**		
Yes	52	12.5%
No	363	87.5%
**Legal history**		
Yes	7	1.7%
No	408	98.3%
**Mental illness**		
Yes	2	0.5%
No	413	99.5%
**History of psychological/physical/sexual violence**		
Yes	24	5.8%
No	391	94.2%
**Family history of substance use**		
Yes	104	25.1%
No	311	74.9%
**History of current substance use**		
Yes	16	3.9%
No	399	96.1%

### Cognitive disorder

The mean score of the Mini-Mental State Exam among pregnant women is (26 ± 2), 95% (SD = 25.8–26.5). Out of all respondents enrolled in this study, 112 (27%) of them had a cognitive impairment and scored (<24/30).

### The logistic regression analysis result

According to this study result, variables such as strong social support [0.11 (0.03–0.23), *p* < 0.02], orthodox religion followers [0.24 (0.12–0.39), *p* < 0.04], >5,000 Ethiopian birr monthly income [0.28 (0.17–0.48), *p* < 0.02], age >26 years old [1.23 (1.14–2.54), *p* < 0.04], unplanned pregnancy [2.78 (1.45–4.32), *p* < 0.02] and rural residence [3.90 (2.23–7.34), *p* < 0.04] were significantly associated with cognitive impairment at 95% confidence interval and *p* < 0.05 (Table 4).

**Table 4:** Bivariate and multivariate logistic regression analysis of respondents attending antenatal service at Dilla University Referral Hospital, Dilla, 2022 (*N* = 415).

Variable	Cognitive disorder		Crude odds ratio (95%)	*p*-value	Adjusted odds ratio (95%)	*p*-value
	Yes	No				
**Social support**						
Poor social support	34	37	1		1	
Moderate social support	61	81	0.82 (0.46, 1.45)	0.13	0.50 (0.25–1.26)	0.29
Strong social support	17	85	0.22 (0.11, 0.44)	0.01	0.11 (0.03–0.23)	0.02
**Religion**						
Protestant	68	133	1		1	
Orthodox	28	131	0.42 (0.25, 0.69)	0.04	0.24 (0.12–0.39)	0.04
Muslim	16	39	0.52 (0.26,1.06)			
**Marital status**						
Married	213	178	0.29 (0.12, 0.66)	0.56	0.13 (0.05–0.33)	0.77
Unmarried	13	11	1		1	
**Educational status**						
Unable to read and write	17	43	1.83 (0.78,4.33)	0.51	1.41 (0.38–3.24)	0.47
Primary	42	96	2.03 (0.96, 4.28)	0.15	1.85 (0.69–3.93)	0.20
Secondary	25	130	0.89 (0.41, 1.94)	0.12	0.70 (0.36–1.69)	0.19
College and above	11	51	1		1	
**Husband occupation**						
Government	35	68	1.57 (0.97, 2.54)	0.32	1.27 (0.58–2.11)	0.59
Private	77	235	1		1	
**Monthly income**						
<5,000ETB	75	163	1		1	
>5,000ETB	37	140	0.57 (0.36, 0.90)	0.04	0.28 (0.17–0.48)	0.03
**Age**						
<26 years old	60	209	1		1	
≥26 years old	52	94	1.93 (1.24, 3.00)	0.01	1.23 (1.14–2.54)	0.04
**Number of ante-natal care**						
<3 visit	15	63	0.59 (0.32, 1.08)	0.32	0.27 (0.23–1.03)	0.44
≥3 visit	97	240	1		1	
**Number of pregnancies**						
<2	26	95	0.66 (0.41, 1.09)	0.31	0.39 (0.26–1.02)	0.37
≥2	86	208	1		1	
**Family size**						
<3	57	189	1.60 (1.03, 2.48)	0.04	1.14 (0.45–1.79)	0.14
≥3	55	114	1		1	
**Loss of a loved one**						
Yes	20	32	1.84 (1.03, 3.38)	0.05	0.93 (0.69–2.57)	0.10
No	92	271	1		1	
**Family substance history**						
Yes	31	73	1.21 (0.74, 1.97)	0.45	1.15 (0.36–1.23)	0.58
No	81	230	1		1	
**Past history of pregnancy complications**						
Yes	21	38	1.61 (0.91, 2.89)	0.52	1.34 (0.35–1.66)	0.60
No	91	265	1		1	
**History of abortion**						
Yes	11	36	0.81 (0.4, 1.65)	0.65	0.62 (0.13–2.45)	0.78
No	101	267	1		1	
**Planned pregnancy**						
Yes	75	269	1		1	
No	37	34	3.9 (2.29, 6.64)	0.01	2.78 (1.45–4.32)	0.02
**Residence**						
Urban	100	296	1		1	
Rural	12	7	5.07 (1.94, 13.24)	0.00	3.90 (2.23–7.34)	0.04

COR, crude odds ratio; AOR, adjusted odds ratio; 1 = reference category; *χ*^2^ = 17.74; Hosmer Lemeshow goodness-of-fit 0.43, degrees of freedom = 8.

## Discussion

Cognitive dysfunction is characterized by deficiencies in motor functioning, linguistic and nonverbal learning, short-term and working memory, visual and auditory processing, problem-solving, and attention (16). According to previous reports, the stress of pregnancy and the influence of hormonal changes during pregnancy may be to blame for reported cognitive impairment (4). This study found that 112 (27%) of respondents had cognitive impairment and variables such as strong social support, orthodox religion follower, unable to read and write, >5,000 Ethiopian birr monthly income, age >26 years old, unplanned pregnancy, and rural residence were significantly associated with cognitive impairment. This study revealed that 27% with 95% CI (22.9%–31.6%) respondents had cognitive impairment which was in line with a study done in northern India, (23.73 ± 4.69) (8). However, this study finding was lower than the studies done in Indonesia (81%) (9), and Nigeria (58.3%) (10). This might be attributed to the difference in the study setting, socio-demographic characteristics, and nutritional status of respondents. This study also revealed that respondents who were orthodox religion followers were 0.24 (0.12–0.39), *p* < 0.04) times less likely to develop cognitive impairment which is consistent with the results of study completed in Australia (20). Perhaps religious activities such as singing, praying, worshipping, attending sermons, bible reading, and socializing with other believers can maintain abundant neocortical brain synapses and minimize cognitive impairment.

Pregnant women who had an income ≥5,000 ETB were 0.28 (0.17–0.48) times less likely to have cognitive impairment than their referent group. This result finding is consistent with the results of other research conducted in Ethiopia (21). An increased monthly income can make it possible for the fulfillment of provisions such as food, shelter, and other necessities that are vital for cognitive development. This study also found that those respondents with age ≥26 years old were 1.23 (1.14–2.54) times more likely to confront cognitive impairment. This is also supported by another study done in Ethiopia (22). Effectiveness on mental tasks, such as tests of processing speed, working memory, and executive cognitive function, which call for one to quickly analyze or change information in order to make a choice, declines with age (23). This study also found that respondents with unplanned pregnancy status were 2.78 (1.45–4.32) times more likely to experience cognitive impairment. Unwanted pregnancies result in psychological ill-preparedness, a lack of interest in the pregnancy period, a lack of motivation to work, excessive worry about the future, and poor assertiveness, all of which are linked to the cognitive deficit (24). This study also found that respondents with rural residence were 3.90 (2.23–7.34) times more likely to encounter cognitive decline as compared with their counterparts. This study finding is consistent with a study done that attributed the impact of living in rural area to a lack of exploring calendars, urban environments, hospitals, and other locations that are necessary for cognitive assessment (25). The final predictor variable associated with cognitive decline was strong social support and this study revealed that respondents with strong social support were 0.11 (0.03–0.23) times less likely to have scores associated with cognitive decline as compared with those with poor social support. Good social support helps with physical health, food, psychological wellbeing, maintaining relationships, practical assistance, emotional support, and confirmation of values and attitudes (26).

## Conclusion

This study found that nearly one-fourth of respondents had developed cognitive impairment during pregnancy. Predictors associated with a higher chance of cognitive impairment were strong social support, following orthodox religion, ≥5,000 Ethiopian birr monthly income, age >26 years old, unplanned pregnancy, and rural residence. In addition, strengthening social support and having a wanted pregnancy is also important good mental state. It is crucial to assess respondents' cognitive capacities in order to provide better psychological antenatal care and to strive to increase social acceptance of their pregnancy. The routine assessment of cognitive function and integrating mental health services with antenatal care service is vital for the overall wellbeing of a pregnant mother.

## Data Availability

The original contributions presented in the study are included in the article, further inquiries can be directed to the corresponding author.
